# Perceptions of health and coping strategies among temporary migrant workers in East and Southeast Asia: a systematic review

**DOI:** 10.1186/s12939-023-01840-7

**Published:** 2023-02-15

**Authors:** Margo Turnbull, Tiffany Ching, Carol Yu

**Affiliations:** 1 International Research Centre for the Advancement of Health Communication, The Hong Kong Polytechnic University, Hung Hom, Kowloon, Hong Kong, China; 2 The Hong Kong Polytechnic University, Hung Hom, Kowloon, Hong Kong, China

**Keywords:** Migrant workers, East Asia, Southeast Asia, Temporary migration

## Abstract

**Background:**

The rate of international migration for the primary purpose of employment has increased exponentially in recent decades. A significant proportion of this global movement takes place across East and Southeast Asia as workers move on a temporary basis from lower-middle-income home countries such as Indonesia, the Philippines, Thailand and Vietnam to high-income host destinations including Hong Kong and Singapore. Relatively little is known about the unique and long-term health needs of this heterogeneous group of people. This systematic review presents an analysis of recent research into the experiences and perceptions of health of temporary migrant workers in the East and Southeast Asian regions.

**Methods:**

Five electronic databases CINAHL Complete (via EbscoHost), EMBASE (including Medline), PsycINFO (via ProQuest), PubMed and Web of Science, were systematically searched for qualitative or mixed methods, peer-reviewed literature published in print or online between January 2010 and December 2020. Quality of the studies was assessed using the Critical Appraisal Checklist for Qualitative Research published by the Joanna Briggs Institute. Findings from the included articles were extracted and synthesised using qualitative thematic analysis.

**Results:**

Eight articles were included in the review. Findings from this review indicate that multiple dimensions of workers’ health is impacted by the processes of temporary migration. In addition, the research reviewed indicated that migrant workers used various strategies and mechanisms to attempt to address their health-related issues and to take better care of themselves. Such agentic practices could help them manage and maintain their health and wellbeing across physical, psychological and spiritual dimensions within the structural constraints of their employment.

**Conclusions:**

Limited published research has focused on the health perceptions and needs of temporary migrant workers in East and Southeast Asia. The studies included in this review focused on female migrant domestic workers in Hong Kong, Singapore, and the Philippines. These studies provide valuable insights but do not reflect the heterogeneity of migrants moving within these regions. The findings of this systematic review highlight that temporary migrant workers experience high and sustained levels of stress and are exposed to certain health risks which may compromise long-term health outcomes. These workers demonstrate knowledge and skills in managing their own health. This suggests that strength-based approaches to health promotion interventions may be effective in optimising their health over time. These findings are relevant to policy makers and non-government organisations supporting migrant workers.

## Introduction

The rate of international migration for the primary purpose of employment has increased exponentially in recent decades. In 2021, the International Labour Organisation (ILO) estimated that there were 169 million migrant workers globally [1]. For many of these individuals, migration to host destinations is motivated by the availability of higher wages and the potential to improve the financial security of themselves and their families [2]. According to the United Nations (UN), migration within the economically and culturally diverse East and Southeast Asian regions accounted for 7% of this global movement in 2015 (UN, 2015 cited in [3]). A considerable proportion of this regional movement is characterised by the flow of workers from lower-middle-income home countries such as Indonesia, the Philippines, Thailand, and Vietnam to high-income host destinations such as Hong Kong and Singapore [4].

A majority of these workers are employed in the host destinations on short-term contracts linked to a single employer and thus meet the international definition of temporary migrant workers [5]. These temporary migrants are often employed to fill workforce gaps in low-skilled jobs across construction, cleaning, and domestic care settings [6]. Increasingly in host destinations including Hong Kong and Singapore, female migrant workers are being employed as private carers for older people living in the community [7, 8]. This trend is predicted to increase as some governments actively promote the provision of private care to help cope with the pressures of ageing populations [4]. In addition to providing valuable services in host destinations, migrant workers contribute to the Gross Domestic Product (GDP) of their home countries by sending significant amounts of money to their families in the form of financial remittances [9, 10].

These patterns of temporary migration have become embedded within Asian labour markets with varying degrees of regulation [3, 9, 11–14]. Although there is increasing interest in the intergenerational impact of this migration [4, 15], relatively little is known about the unique and long-term health needs of the individuals who make up this heterogeneous group [16, 17]. Workers are required to undergo health screening before departing to start new work contracts and may therefore be in relatively sound physical health at the time of employment [17]. However, the cumulative impact of long-term exposure to health risk factors [17] including familial poverty [18], family separation, limited workplace protections and difficulties negotiating unfamiliar health systems predisposes these individuals to complex physical and psychological health problems [9, 16, 19, 20].

This systematic review contributes novel insights into how workers in the East and Southeast Asian regions conceptualise and talk about their health and coping strategies through stages of temporary migration and within the structures of their employment. In this review, we take a broad view of health aligned with recent extensions of Atonovsky’s multi-dimensional salutogenic model [21–24]. This work considers health in terms of the activation of individual strengths and resources across bio-psycho-social dimensions. Health is expressed through an individual’s ability to adapt to and cope with challenges and stressors. This is supplemented by the critical and praxeological work of Dutta [25–28] and Samerski [29] who recognise health as (re)produced across multiple contexts and domains. Importantly, this critical viewpoint moves away from a pathogenic view of health focused on injury and disease [22, 24] and which has been the dominant approach taken in research with migrant workers. In alignment with these perspectives and to reflect a period of significant regional policy development and increased rates of migration [3], this review included qualitative and mixed methods research published between 2010 and 2020. This review is a timely contribution to the literature as existing health inequities and challenges affecting migrant workers have been exacerbated by the COVID-19 pandemic [30].

## Methods

This systematic review was registered prospectively with PROSPERO (CRD42021286062). Findings are reported in accordance with Preferred Reporting Items for Systematic Reviews and Meta-Analyses (PRISMA) guidelines [31] and with the recommended reference to the statement of Enhancing Transparency in Reporting the Synthesis of Qualitative Research (ENTREQ) [32].

### Inclusion and exclusion criteria

Inclusion criteria are summarized in Table 1.

**Table 1:** Inclusion criteria

**Criteria**	**Inclusion**
Type of literature/data	Original, empirical research articles
Publication type	Peer-reviewed journal articles
Year of publication	Online or in print between January 2010 and December 2020
Type of studies	Qualitative or mixed methods studies
Location of data collection	Data collected in either the home or host nations of the workers within four key locations in East and Southeast Asia (Hong Kong, Indonesia, Singapore, the Philippines)
Target population	Passport holders from one of the following nations: Indonesia, Malaysia, Singapore, Thailand or the Philippines. Research participants also had to be adult migrant workers (aged over 18 years) who had worked in paid employment gained through legal recruitment methods in the East or Southeast Asian regions
Research focus	Perceptions and experiences of adult migrant workers in terms of physical, psychological and spiritual health

Articles were excluded if they were related to undocumented or illegally employed migrant workers, internally migrated/displaced people, refugees, families of migrant workers and migrant workers under the age of 18. Studies which reported only on clinically diagnosed conditions (e.g., diabetes) or workplace accidents were also excluded. It should be noted that while studies related to the COVID-19 pandemic were identified in database searches, they were excluded from the review.

### Searches

This systematic review was undertaken between February and August 2022. Five electronic databases CINAHL Complete (via EbscoHost), EMBASE (including Medline), PsycINFO (via ProQuest), PubMed and Web of Science, were systematically searched for relevant peer-reviewed literature published either online or in print between January 2010 and December 2020. This period was selected as it covers a time of significant regional policy development and increased rates of migration [3]. Geographical locations were chosen based on data about current migration patterns and government visa programmes which facilitate this inter-regional flow of workers [14]. No language restrictions were applied to the searches. Search algorithms are shown in Table 2.

**Table 2:**
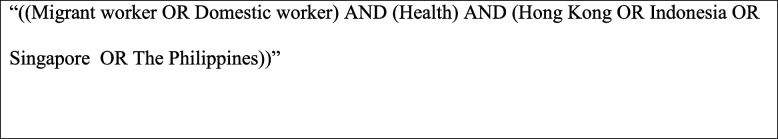
Search terms

In this systematic review, all stages of screening of studies were conducted manually. Initial searches returned 1,302 articles. Five articles were in languages other than English. After removing duplicates and non-peer reviewed publications (e.g., news articles), 808 titles were reviewed against the inclusion criteria by two authors (MT and TC). At this stage, the articles in languages other than English were excluded as the research described was conducted outside the East and Southeast Asian regions (e.g., one in Argentina and two in both Norway and the Republic of Korea). After further exclusions, 123 abstracts were reviewed by MT and TC. Thirty-nine articles were then downloaded for full text review. All authors (MT, TC and CY) were involved in the full text review stage. Each article was initially evaluated for its eligibility by one of the three authors based on the inclusion and exclusion criteria. The final decision on inclusion/exclusion was made after consensus was reached by all three authors. After full text review, eight articles were included in the data synthesis. The reference lists of these eight articles were manually searched for additional inclusions. No additional articles for inclusion were identified through this process.

### Study quality assessment

The Critical Appraisal Checklist for Qualitative Research published by the Joanna Briggs Institute [33] was used to rate the strength and quality of the eight eligible studies. Each eligible study was assessed by one of the three authors independently and the final decision to include/exclude was made through discussion with all authors. All articles selected for inclusion in the systematic review were appraised as being of high quality across the domains of congruity between methodology and methods, data representation and interpretation, ethical approval and informed consent, representation of participants voices and researcher positioning.

### Data extraction strategy

Information was extracted from the Findings/Results and Discussion sections of the included articles and stored in a Microsoft Excel file. Data extracted related to (a) aim(s), (b) type of study, (c) location of data collection, (d) participants, (e) sample size, (f) data collection method(s), (g) analytical approach and (h) key findings.

### Data synthesis and presentation

Qualitative thematic analysis [34] was used for synthesis of the studies included in the review. Each of the included studies was inductively coded independently by two of the three authors. The initial stage involved line by line reading and data extraction. After group discussion, a final framework that drew on relevant theories [25–27, 29] was developed. Descriptive themes generated by all authors were grouped into codes. Relevant quotes from research participants were also extracted to further substantiate theme development. Coding was discussed at each stage by two authors. Any disagreements were resolved by inclusion of the third author. Final coding was discussed and agreed by all authors.

## Results

**Fig. 1:**
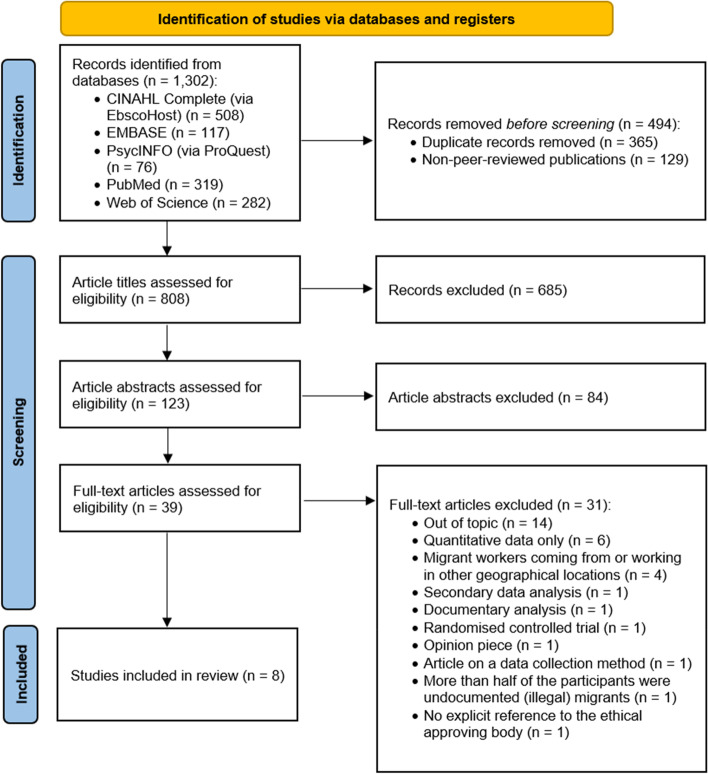
PRISMA flow diagram [31] of included and excluded studies of the systematic review

Eight articles published online or in print between January 2010 and December 2020 were included in the review (one article [35] has an in-print publication date of 2021 but was published online in 2020). A summary of search results is shown in Fig. 1. Despite the broad search terms that included a range of geographical locations, work types and genders, all articles that met the inclusion criteria were based on data collected in Hong Kong, Singapore and the Philippines, and focused exclusively on the experiences of female migrant workers employed in domestic settings. There was variation across articles in terminology used to refer to these workers (either Foreign Domestic Workers or Migrant Domestic Workers). For consistency, in this article we use the term Migrant Domestic Workers (MDWs). It is of note that two articles [28, 35] drew on data collected in the same longitudinal study conducted in Singapore. Both articles were included in the review as they focused on different aspects of the research. Similarly, two articles [6, 36] drew on the same large research project focused on data collected in the Philippines. Both articles were also included as they reported on different aspects of the research.

Six articles [7, 8, 20, 28, 35, 37] reported on data collected from MDWs during a period of employment in the host location, either in Hong Kong [20] or Singapore [7, 8, 28, 35, 37]. The other two included articles [6, 36] draw on data collected from research participants after their return to their home country (the Philippines). Most research participants were reported as being from the Philippines. Indonesian workers were the next most represented national group. A small number of research participants were from Myanmar and Sri Lanka. These details are summarized in Table 3.

**Table 3:** Data extraction table

**Author(s)**	**Aim(s)**	**Type of study**	**Location of data collection**	**Participants**	**Sample size**	**Data collection method(s)**	**Analytical approach**	**Key findings**
Bernadas and Jiang [20]	To explore and problematized the reasons, processes, and barriers in acquiring health information among Filipino MDWs in Hong Kong	Qualitative	Hong Kong	Female MDWs from the Philippines living in Hong Kong, aged between 27 and 62 with at least 24 months work experience	23	Four focus group discussions	Grounded Theory	• Filipino MDWs in Hong Kong sought more treatment than prevention-related information. Information seeking was characterized as sequential across multiple sources • Various information sources were identified, including health care professionals (e.g., doctors or nurses) and nonprofessional sources (e.g., employers, family, or friends) and the Internet (e.g., search engines) • Barriers to information seeking included structures of work and immigration • Unknown quality of information accessed was also a potential barrier to health decision-making • Religiousness, including beliefs and attendance at church, motivated health-seeking behavior and increased access to sources of health information
Dutta et al. [28]	To explore the key meanings of health held by MDWs in Singapore as they negotiate living and working in the country	Qualitative	Singapore	Female MDWs with at least two months work experience	20	Semi-structured interviews	Culture Centered Approach	• Food and financial insecurity were identified as key structural features that influenced the health of MDWs in Singapore • Findings described how MDWs participated in making health choices • MDWs described themselves as making choices about their daily health through agentic actions such as exercising while doing chores, drinking water, self-medicating, participating in communal activities and praying
				Interviewees were residing in non-government shelters after leaving abusive employment situations				
Heng et al. [8]	To explore the caregiving experiences and coping strategies of MDWs workers caring for frail older people in Singapore	Qualitative	Singapore	Female MDWs aged between 26 and 42 years with between 2 months and 13 years of work experience with older people	11 Participants were from the following home countries: Indonesia (*n* = 6), the Philippines (*n* = 4), and Myanmar (*n* = 1)	Semi-structured interviews	Thematic analysis	Four main themes were identified: 1. MDWs had to balance attending to the care needs of their employers as well as completing household cleaning duties. Striving to achieve this balance created significant stress 2. MDWs encountered challenges caring for the older, frail care-recipients. Language barriers may lead to miscommunication. Workers faced challenges in handling behavioral issues of their carerecipients. Workers reported not having enough rest, and they had concerns about leaving their care-recipients alone or letting go of their caregiving responsibilities on their days off 3. Workers used various strategies to help themselves cope with stress, including time management strategies, emotional coping strategies such as treating their care -recipients as their own family members, and self-distraction such as playing games on social media platforms, singing and dancing 4. Workers sought emotional support from their employers, family and friends. MDWs sought training and nursing skills from healthcare professionals
				MDWs were employed to provide care to individuals aged over 60 who needed assistance in at least one activity of daily living				
Kaur-Gill and Dutta [35]	To represent voices of MDWs in the exploration of their everyday mental health	Qualitative	Singapore	Female MDWs with at least two months work experience and residing living in temporary emergency accommodation. Interviewees had left abusive employment situations	20	In-depth interviews	Culture Centered Approach	MDWs described careful negotiation of their highly structured and monitored lives in their employers’ homes
								Through narratives, MDWs illustrated how the structural context of their employment affected their mental health
Tam et al. [7]	To explore MDWs' perceptions about the challenges, coping strategies, and support needed to care for frail seniors	Qualitative	Singapore	Female MDWs from Indonesia, the Philippines, and Myanmar living in Singapore, aged between 21 and 50 years with at least 12 months work experience looking after frail, older people	25	In-depth interviews	Thematic analysis	This study identified three main themes related to factors that both challenged and supported MDWs employed to care for frail, older people: 1. Social support and positive relationships with both the employer and their extended family 2. Access to outside social circles and adequate weekly rest days 3. Job satisfaction derived from the act of caring which was linked with perceptions of self-efficacy and skills and knowledge about specific needs of the elderly employer (e.g., dementia)
				Five full-time healthcare staff of the hospital where data was collected				
Van Bortel et al. [37]	To examine stressors and coping mechanisms experienced by female MDWs in Singapore	Qualitative	Singapore	Female MDWs living in Singapore, aged between 20 and 63 years with at least 12 months work experience	100 of 182 questionnaire respondents responded to free-text questions which were analyzed qualitatively	Free-text responses gathered through a cross-sectional survey	Thematic analysis and content analysis	Three themes were identified as key underlying causes of stress: 1. Work restrictions and lack of personal agency and control, 2. The pervasiveness of financial need, and 3. Commitment to families and associated obligations
								Coping strategies described by MDWs were grouped into three areas: 1. Finding time for self, 2. Managing thoughts, and 3. Engaging in religious activities
					Participants were from: the Philippines (*n* = 104), Indonesia (*n* = 68), Myanmar (*n* = 9), and Sri Lanka (*n* = 1)			
								Social support was an additional protective factor
Van der Ham et al. [6]	To describe and explore factors that relate to stress, resilience and wellbeing for female MDWs from the Philippines	Mixed methods	The Philippines	500 female MDWs who had returned to the Philippines after completing at least one work contract abroad	500	Questionnaire with open and closed-ended questions administered orally through structured interviews	This publication describes using content analysis for analysis of data from workshops and focus groups	• Participants reported that they experienced high levels of stress but perceived their wellbeing during migration as generally good • This study explores two factors that might support resilience among Filipino MDWs: (1) personal resources (reasons for migration, coping) and (2) social resources (employer-worker relationship, social support, organization membership) • Most of the reasons for migration given by the participants were related to escaping poverty and improving quality of life • Participants used various coping strategies to deal with stress. Praying/reading the Bible, crying, resting/sleeping were the most frequently used ways • Participants reported spirituality (especially praying) as a source of strength for them • Some participants reported positive experiences with employers, for example, receiving material gifts and being praised for job well done • Filipino friends in the country of destination and family members in the home country (the Philippines) were also important sources of social support
				Research participants were aged between 18 and 60 years and had worked in countries in Asia and the Middle East during their last contracts				
						Workshops with 23 MDWs and representatives from governmental organizations (*n* = 7) and non-governmental organizations (*n* = 13), and field interviewers (*n* = 3)		
						21 MDWs in two focus groups (*n* = 13; *n* = 8)		
Van der Ham et al. [36]	To assess stress and coping of female MDWs from the Philippines across three different phases of migration (pre-, during- and post-migration)	Mixed methods	The Philippines	500 female MDWs who had returned to the Philippines after completing at least one work contract abroad	500	Questionnaire with open and closed-ended questions administered orally through structured interviews	Responses to free-text questionnaire items were analyzed thematically	• MDWs experienced higher levels of stress during migration than when they were in the Philippines (either prior to or after migration) • Stressors identified by MDWs while they were in the Philippines were related to financial issues, while key causes of stress during migration were linked more strongly to loneliness, homesickness, poor working conditions, and employment conditions
				Workers were aged between 18 and 60 years and had worked in countries in Asia and the Middle East during their last contracts				
						Workshops with 23 MDWs and representatives from governmental organizations (*n* = 7) and non-governmental organizations (*n* = 13), and field interviewers (*n* = 3)		
						21 MDWs in two focus groups (*n* = 13; *n* = 8)		

Thematic analysis of the eight articles highlighted two core themes: (1) health and migration; and (2) health as negotiation of agency and structures via agentic physical, psychological and spiritual (faith-based, religious) practices. These themes and associated subthemes are summarised in Table 4 and are discussed in detail in the following sections.

**Table 4:** Summary of themes and subthemes

**Themes**	**Subthemes**	**Studies included**
Health and migration	Pre-migration health	Van Bortel et al., [37]; Van der Ham et al., [6, 36]
	Health during migration	Heng et al., [8]; Dutta et al., [28]; Kaur-Gill & Dutta [35]; Tam et al., [7]; Van Bortel et al., [37]; Van der Ham et al., [6, 36]
	Health after a period of migration	Van der Ham et al., [36]
Health as negotiation of agency and structure (agentic practices)	Physical self-care	Dutta et al., [28]
	Psychological self-care	Bernadas & Jiang [20]; Dutta et al., [28]; Heng et al., [8]; Kaur-Gill & Dutta [35]; Tam et al., [7]; Van Bortel et al., [37]; Van der Ham et al., [6, 36]
	Spiritual self-care	Bernadas & Jiang [20]; Dutta et al., [28]; Heng et al., [8]; Van Bortel et al., [37]; Van der Ham et al., [6, 36]

### Theme 1: Health and migration

Seven of the articles included in this review explicitly focused on the psychological (mental) health of MDWs [6–8, 28, 35–37]. The other study [20] explored the health-seeking behaviours of MDWs in Hong Kong and included supplementary discussion of psychological health. Within the eight included studies, physical health was discussed in terms of the capacity to perform job tasks and to contend with factors associated with the structures of employment, such as restricted access to food [28], lack of rest and physical exhaustion [36], injury [20] or physical abuse [35]. It is of note that psychological health was not discussed in terms of impairment or diagnosed disorders, but rather in ways that emphasised the cumulative impact of long-term and high levels of stress that were often derived from the complex relationships MDWs navigated with both their families and employers. The sources and manifestations of psychological stress reflect the multiple stages of movement between home and host destinations that characterise this temporary migration. This variation in stress and psychological health across the migration process was highlighted by Van der Ham et al. [6, 36] in their study of MDWs who had returned to the Philippines. These authors found distinctions in sources of stress across three phases of migration: pre-migration, during migration and post-migration.

#### Pre-migration health

As previously noted in this article, although temporary migrant workers are required to have pre-employment health screenings, exposure to risks associated with poverty and stress linked with family separations may influence long-term health outcomes [5]. In the literature reviewed, pre-migration health of MDWs was discussed in terms of psychological stress associated with concerns about leaving their families and becoming migrant workers to alleviate poverty and provide financial support to their families [6, 33, 37]. Given that financial concerns often drove migration, individuals were likely to enter the migration process with high levels of stress and anxiety and having been exposed to health risk factors such as poor nutrition associated with familial poverty. As noted by Van der Ham et al. [6], the migration of women particularly into roles as domestic workers “has increasingly become a structural survival strategy for migrants and their families” (p. 546). These workers are often driven by the goals of “[e]scaping poverty and improving quality of life” ([6], p. 563).

#### Health during migration

MDWs’ narration of their health indicates that during a period of migration, their health was influenced by the intersection of individuals’ work context and the complex bonds that connected them to their families in their home countries. Once the MDWs had arrived in host destinations and started working, they reported positive emotions as a result of being able to provide for the  everyday needs of their families as is reflected in the following comment made by an MDW:

*You feel good thinking that your family can eat whatever they like at least once a week* ([6], p. 558).

Families were both a source of psychological stress as well as a vital coping mechanism for some MDWs [6]. The complexity of these relationships is visible in the description of MDWs as both carers and breadwinners for their families [37]. Health for MDWs was intertwined with their ability and desire to achieve financial security for themselves and their families [6, 28, 36]. This suggests that during employment, high levels of psychological stress are often maintained due to the geographic distance from families and continued financial pressures [28, 37]. The combination of being away from families as well as the burden of financial responsibilities was linked with feelings of loneliness, homesickness and isolation from their social networks and cultures [8, 36, 37]. MDWs also reported that they experienced a higher level of psychological stress when they thought about the circumstances in which their loved ones lived at home [37].

Psychological health was also impacted by stress and anxiety attributed to the navigation of familial pressures and those associated with MDWs employment [6, 37]. This is illustrated in the following comment:

*[I feel stressed] when I can’t reach someone at home and I can’t call. . .When loved ones demand time that I could not give due to working (participant 120)* ([37], p. 8).

For some MDWs, financial pressures were exacerbated once they started working as they had to pay off agency debts after arriving in the host destination [28, 35]. Other causes of stress in host destinations also included fear of losing their jobs [35], restrictions on visas and the way visas were tied to employers [37]:

*[What is stressful is] my situation today, losing my job and looking for another employer (participant 132)* ([37], p.7).

For others, the inherent inequality of the relationship between them and their employer was a cause of significant stress:

*Sometimes my duty to serve my employer’s family make me feel stressed (participant 103)”* ([37], p. 5).

*When my employer gets mad, I really wanted to talk back but I didn’t because I was afraid to get fired. I just endured it* ([9], p. 556).

The deliberate manipulation of this imbalance by some employers directly impacted upon both physical and psychological wellbeing of workers. This is evident in a narrative told by an MDW in Singapore of not being given enough food:

*She didn’t give enough food, and I was always hungry. She gave me rice one week one time. After that, I mopped all the house with my hands, and I felt tired and hungry. And then, after, after that, my employer Ma’am, I said, can I take the one bread? She said no. The child, the Ma’am’s child, she said can I give her the one piece of bread. She [referring to the employer] said no, you must drink only water. You must wait what time you eat. And then after that, my hands and whole body were shaking* ([28] p. 646).

The nature of employment tasks was also a cause of stress for some MDWs and was linked to perceived burdens of work and burn-out [7], social isolation in the host countries [36] and the time-consuming nature of the work itself [6]. Caring for older people was discussed as particularly stressful for migrant workers as the work often involved attending to daily living needs such as personal hygiene and feeding [7, 8]. Some research participants believed that these caring tasks required training, which was not available to them, and thus they felt insecure in their ability to provide adequate care:

*I must drain the urine, use the indwelling catheter ... I transfer him... I do the dressing...because there are some wounds... I give medicine before breakfast... Then I just think whether I can or not. Because I am not a nurse, original nurse, I haven't study... (P06)* ([8], p. 461).

The accumulation of psychological and emotional stress increased the vulnerability of some MDWs to physical, verbal, sexual and racial abuse, and exploitation [35, 37]. In these studies, some MDWs reported that they tolerated poor working conditions and abusive (illegal) treatment by employers because of the risk of being fired if they complained. Narratives detailed heavy workloads and insufficient rest [36, 37], lack of food [28, 35, 36] and insufficient space and privacy [35].

The psychological impact of poor treatment in the workplace was exacerbated for some MDWs who could not find sources of support [35]. This is evident in the interview extract below in which a MDW describes unsuccessfully seeking help from the employment agent who found the job for her:

*At 1st 3 months … months, I talked to my agent. I told them everything. What for your skin burnt, You stupid, You chicken brain. Like that, I told to my agent. BUT HE DON’T BELIEVE ME [expressed this loudly] … I think he don’t believe me, cos I told him if I can transfer. BUT he just told me ‘better to go home (back) to your ‘poor’ country’. If it’s really difficult for you* ([35], p. 1471).

Psychological stress was identified as a significant and ongoing concern for MDWs and was linked with negative emotions and symptoms of depression or anxiety such as sadness [35], crying [6, 28, 36] or sleeplessness [7].

#### Health after a period of migration

One article included [36] offered insights into the psychological health of MDWs once they returned to the Philippines after a period of migration. Van der Ham et al. [36] observed that post-migration stress was connected with re-adjustment to the home nation and the individual family context. MDWs often returned home to face ongoing financial stress which prompted individuals to take up more work in foreign countries, and thus embark on another cycle of temporary migration.

### Theme 2: Health as negotiation of agency and structure (agentic practices)

The analysis of the articles included in this review highlights that despite the complexity of the factors that influenced MDWs health, individuals connected their health to their capacity for agency in their daily lives. This notion of health through agency (agentic practices) resonates with Dutta’s [25] observation that health can be viewed as the manifestation of individuals’ conscious decision making within the structural constraints of their everyday environment. Expressions of health were described in terms of self-care and involved negotiation of constraints associated with locations and conditions of employment. The negotiation led to the enactment of health across three dimensions – physical, psychological, and spiritual.

#### Physical self-care

Dutta et al. [28]’s study on health meanings among MDWs in Singapore found that MDWs engaged in various practices to address their physical health needs within structural constraints. Research participants discussed the notion of “living well” (p. 649) as personal responsibility:

*Because I am the one who can manage myself regarding the foods and the detergents, I should be careful (Anne)*

*You care for yourself. You are the responsible one for yourself. That is the first thing. Then, especially the personal hygiene here is very good. A lot of water. And the ambiance is good. This is only about how you care about yourself (Alice)* (p. 648).

The idea of “living well” was manifested in everyday practices such as maintaining good personal hygiene, exercising, eating nutritious foods, getting enough sleep, drinking water and self-medicating. These agentic health practices illustrate how MDWs negotiated their health amid structural constraints. For instance, it was found in Dutta et al. [28]’s study that food security (food adequacy, cultural appropriation and buying choices) was one of the health challenges encountered by the MDWs in Singapore. Despite their limited control over food security, MDWs found ways to maximise their nutrition intake by eating more fruits and vegetables, drinking more water instead of coffee and avoiding greasy food. Below, a MDW shared how she expressed her agency in modifying her dietary practices to keep herself healthy:

*… healthy is, if must exercise, then can be healthy. Must eat food also, must eat healthy food … no so many, so much oil. Sometimes, can eat rice, veggie and fruits, milk, like that. Don’t so many eat ah coffee, don’t so many eat oil, and must do exercise … and can be healthy and can be fit* ([28], p. 648).

For many MDWs, the nature of their job meant that they were confined to the house of their employers most of the time. Nevertheless, MDWs described how they incorporated exercise into daily activities such as climbing stairs and dancing while doing household chores [28]. MDWs also described enacting their agency in health in terms of finding ways to treat their own health needs [28]. This study also reported that interview participants preferred self-medication as it was more accessible and that they regarded homemade remedies as more effective for treating health problems such as headaches, stomach-ache and body pain.

#### Psychological self-care

Aspects of stress and psychological health of MDWs were discussed in all studies included in this systematic review [6–8, 20, 28, 35–37]. Van der Ham et al. [36] found that emotion-focused coping styles became dominant during migration (as compared to more problem-focus coping styles in the pre-migration stage). A good support structure made up of employers, families, other MDWs and healthcare professionals was described as vital to the facilitation of psychological self-care [6, 8, 28, 35, 37]. Regular communication with families in home nations provided MDWs with emotional support [6, 8, 35] and was linked with relieving stress and supporting perseverance through difficult experiences [6, 8]. During their employment in host regions, MDWs made extensive use of formal and informal group memberships to establish interpersonal relationships and to provide and access social support [6, 28]. Religious and cultural groups, non-government organizations (NGOs) for overseas domestic workers and women, volunteer groups and sexual identity groups provided important opportunities for MDWs to establish networks outside of their employment context [6, 28]. In addition to providing emotional support, friends and churches were also identified as useful sources of health information. Social networking sites were found to help promote health awareness among communities of MDWs and also facilitated the dissemination of health information [20].

Apart from seeking emotional support from social resources, MDWs also employed personal resources for psychological self-care. Laughing, joking, singing or crying [8, 28, 36], taking time for self [8, 36, 37], positive self-talk [7], praying and reading the Bible [36], playing games on social media platforms [8] were identified as psychological coping strategies by MDWs. These are reflected in the comments of participants shown below:

*I sing … If they [employers] are not in the house, I sing loud, loud. Like I cry also.”* (Sandy)

*[I cope by] just crying, then sometimes I tell myself, no, I don’t want to cry because I know I can handle this (Mim)* ([28], p. 650).

In terms of seeking strength from within, thinking positively and focusing on the present were reported as psychological coping strategies employed by the MDWs in Van Bortel et al.’s [37] study. Similarly, Van der Ham et al. [6] found that the capacity to endure and accept the situation was described as an important coping strategy by MDWs. The authors argued that these passive emotion coping strategies reflected the MDWs’ marginalized and isolated positions and their lack of control over their work conditions, which forced them to find sources of strength internally.

In addition to passive coping strategies, Tam et al. [7] described having a sense of self-efficacy in their job as a stress mitigating strategy which could improve MDWs’ wellbeing. Developing job-related skills such as learning the language of the care-recipients was regarded as crucial by MDWs to enabling better care for their care-recipients [6, 8]. Massage and hairdressing skills were found to be useful for MDWs who were employed to care for people with dementia [7]. These strategies not only helped MDWs develop a sense of self-efficacy in their employment but also encouraged more trust and better relationships with their employers and/or care-recipients. This was considered vital to the MDWs overall wellbeing as the employer was regarded as the “central structural actor” ([35], p. 1468) who could provide them with both support and respect [6–8, 35].

#### Spiritual self-care

MDWs also discussed spiritual self-care practices which were linked to their psychological wellbeing [6, 8, 20, 28, 36, 37]. Spiritual self-care was linked with MDWs religious faith and associated practices. Related agentic practices included praying, upholding their commitment to and having faith in God as well as participating in social activities organised by faith-based organisations [6, 8, 20, 28, 36, 37]. Van der Ham et al. [36] reported religious activities as the most common coping strategies used by Filipino MDWs during migration. Extracts from Van Bortel et al.’s study [37] below illustrate how MDWs found peace of mind through their faith in God:

*Casting all my anxieties and cares to God help me relax (participant 100)*

*Yes. Because I feel the comfort of God* *(participant 14)*

*Praying clear my mind and refresh my inner thoughts (participant 165)* (p. 9).

Research participants in Dutta et al.’s [28] study described their health as inseparable from their faith in God. They discussed spiritual health as linked with having faith in God and connected this to dimensions of physical and psychological health. Participants also found peace and strength in God and felt that praying helped them cope with sadness and loneliness. One participant described that her religious commitment helped to ease her anger and forgive her employers:

*I am always praying to God that I am thankful that I have my new life once again because that time, I don’t know if I’d live again because of the suffering. And then I already forgive them because what for to keep angry, what for? Because if Lord God can forgive, how you could, how could be me only that could not forgive them? (Anne)* ([28], p. 650)

In their study of MDWs’ information seeking behaviours in Hong Kong, Bernadas and Jiang [20] found that religious engagement not only provided psychological and social resources to promote healthy behaviours of MDWs, but it also facilitated their access to health information. The study highlighted that MDWs could broaden their sources of health information by participating in activities organised by religious organizations [20].

## Discussion

Although the marginalization of migrant workers and associated health impacts are well recognised within the literature [22, 38–40], most of the published research focused on the intersection of temporary migration and health has been conducted in Europe [41, 42], Australia [43, 44] and Canada [45–49]. This systematic review adds to that body of work by analyzing how migrant workers in the East and Southeast Asian regions conceptualize their health and strategies for coping with the impacts of temporary migration. In the research reviewed, health was a fundamental aspect of workers’ lives, and research participants often displayed knowledge and agency in relation to their health across physical, psychological, and spiritual dimensions. However, workers’ agentic health practices were often limited by constraints associated with the processes of migration and structures of employment such as work hours and location, and the control exerted by employers. Research linking health outcomes and migration in other global regions has highlighted the importance of acculturation for health [23]. Workers engaged in cycles of temporary migration occupy a unique and precarious position across both home countries and host destinations. Short-term work contracts and the maintenance of strong links with families as well as the replication of social networks in host destinations are factors which provide great support but may also limit an individual’s acculturation over time.

The importance of agency in terms of health and coping strategies highlighted in this systematic review underscores the need to include migrant workers in the development and delivery of health promotion activities. As noted in recent commentary [50], an inclusive and positive approach can optimise the benefits of “strength-based and solution-oriented” approaches towards health as well as contextualising specific issues (p. 5). Osborne et al. [50] argued that deficit-based models draw attention to what people do not do or lack in terms of skills and knowledge. Such approaches risk increasing marginalization and stigma for certain groups and may elide the complexity associated with contexts such as those created by temporary migration. Health interventions and health promotion programmes incorporating models of strength development (rather than deficit-based models) may be valid ways to improve the overall health of temporary migrant workers.

This systematic review has also highlighted that considerable stress for MDWs is derived from the nature of the work that some of them do. Although it is well recognised that MDWs often take up jobs described as “dirty, dangerous, and degrading” ([6], p. 546), little research attention has been directed towards understanding how the nature of this work may affect individuals’ health outcomes. This is particularly important as some countries across Asia are actively increasing recruitment of MDWs as private carers for older people [4]. Countries that have adopted policies of employing low-paid and low-skilled workers for this complex care work are likely to see poor outcomes for both the older people as well as the migrant workers unless additional investment in specialised training is provided.

### Limitations

The findings of this systematic review are limited in scope by the paucity of literature focused on temporary migrant workers in the East and Southeast Asian regions [3]. Despite broad search terms, the literature available for inclusion was primarily based on research conducted in Hong Kong and Singapore (refer to Table 3). Additionally, all research participants were female, and a majority were from the Philippines. This profile of research participants fails to reflect the heterogeneity of the broader migrant population in these regions. This also indicates that there is limited peer-reviewed research undertaken in significant countries of origin of temporary migrant workers such as Indonesia, Malaysia and Thailand. As noted by Kapilashrami et al. [19] and Sweileh et al. [51], given the volume of migration within the broader Asian regions, these workers are underrepresented within the published literature. This underrepresentation may be partly attributable to the challenges of engaging vulnerable and transient groups in formalised research as well as funding biases which promote greater research activity in high-income locations [19].

Only peer-reviewed literature was included in this systematic review. Future research on the health perspectives and practices of migrant workers could usefully include grey literature and government papers published in a variety of languages in the home nations of migrant workers. As noted by Kapilashrami et al. [19], NGOs play a significant role in supporting migrant health across Asia. Publications by NGOs may also be valuable sources of information for inclusion in such studies.

## Conclusions

Although temporary migration for the purpose of employment drives a significant degree of human movement within the East and Southeast Asian regions, relatively little is known about the health needs and behaviours of this heterogeneous group of people. The analysis of the literature included in this systematic review highlights how health and coping strategies are influenced by temporary migration for female MDWs in East and Southeast Asia. MDWs are situated in precarious positions across social, family and health systems of both their home and host countries and are exposed to significant physical and psychological health risks over time. It is of note that MDWs expressed significant interest, motivation, and skills in maintaining and managing their own health. This suggests that strength-based approaches [50] which leverage strong social networks in host destinations as well as existing knowledge provide optimal avenues for health promotion activities.

## Data Availability

Not applicable

## Abbreviations

ENTREQ: ENhancing Transparency in REporting the synthesis of Qualitative research
GDP: Gross Domestic Product
ILO: International Labour Organisation
MDW: Migrant Domestic Worker
NGO: Non-Government Organization
OFW: Overseas Filipino Workers
PRISMA: Preferred Reporting Items for Systematic Reviews and Meta-Analyses
UN: United Nations
WHO: World Health Organization
